# Acupuncture and Moxibustion for Peripheral Neuropathic Pain: A Frequentist Network Meta-Analysis and Cost-Effectiveness Evaluation

**DOI:** 10.1155/2022/6886465

**Published:** 2022-03-16

**Authors:** Weixuan Zhao, Haoming Huang, Kun Liu, Shuxin Wang, Shiyu Lin, Wenjie Long, Lixia Li, Jingchun Zeng, Guohua Lin

**Affiliations:** ^1^Department of Chinese Medicine, The First Affiliated Hospital, School of Clinical Medicine, Guangdong Pharmaceutical University, Guangzhou 510180, Guangdong, China; ^2^Department of Radiology, The First Affiliated Hospital, Guangzhou University of Chinese Medicine, Guangzhou 510405, Guangdong, China; ^3^Department of Acupuncture, Guangdong Second Traditional Chinese Medicine Hospital, Guangzhou 510095, Guangdong, China; ^4^Department of Acupuncture and Rehabilitation, The First Affiliated Hospital, Guangzhou University of Chinese Medicine, Guangzhou 510405, Guangdong, China; ^5^Department of Geriatric Medicine, The First Affiliated Hospital, Guangzhou University of Chinese Medicine, Guangzhou 510405, Guangdong, China; ^6^Department of Acupuncture, The Affiliated Traditional Chinese Medicine Hospital, Guangzhou Medical University, Guangzhou 510130, Guangdong, China

## Abstract

**Purpose:**

Acupuncture and moxibustion techniques have been increasingly used to treat peripheral neuropathic pain (PNP). However, there is a paucity of comparative information and cost-effectiveness assessment for techniques on PNP management. *Patients and Methods*. Randomized controlled trials studying the acupuncture or moxibustion treatments on PNP were identified from electronic databases. The quality of the included studies and the potential risk of bias was evaluated using the ROB 2.0 assessment tool. The primary outcome was at least 20% pain relief. The treatment effects were pooled through a frequentist-based network meta approach. Subsequently, the cost-effectiveness measured by incremental cost per additional responder (ICPR) was calculated.

**Results:**

One three-arm trial and 15 two-arm trials comprising 1308 participants that satisfy the eligibility criteria were identified. Among the included studies, 12.5% were at low risk of bias, 68.75% had some concerns about the risk of bias, and 18.75% were at high risk of bias. The major sources of bias originated from the randomization processes of the studies. The patients were assigned to seven different acupuncture or moxibustion interventions and two pharmaceutical treatments. Except for acupoint injection, all the included acupuncture and moxibustion techniques showed superior improvements in PNP and were more cost-effective as compared to pharmaceutical treatments. Warm needling, fire needling, and moxibustion were the most effective treatments. Fire needling showed the lowest ICPR relative to the nonsteroidal anti-inflammatory drugs in the cost-effectiveness analysis of direct and indirect costs.

**Conclusion:**

Acupuncture and moxibustion techniques are beneficial and cost-effective approaches for easing PNP and hence can be considered for PNP management.

## 1. Introduction

Neuropathic pain (NP) is a common condition caused by different sources of lesions or diseases underlying the damages to the somatosensory nervous system. The pain is subclassified as peripheral and central based on different pathological and anatomical origins [1]. As for peripheral neuropathic pain (PNP), the inflammatory processes triggered after peripheral nerve lesion together with the substantial release of immune modulators can contribute to peripheral sensitization and nociceptors excitation [2]. According to the advanced classification defined by the International Association for the Study of Pain, chronic PNPs are as follows: trigeminal neuralgia, peripheral nerve injury, painful polyneuropathy, postherpetic neuralgia, and painful radiculopathy [3].

Until recently, there was still lacking reliable information regarding the epidemiology related to PNP or NP [4]]. Some epidemiological studies suggested that the incidence rate of NP was 8.2/1000 person-years (95% confidence interval, CI: 8.0–8.4) [5], and the overall prevalence was 1–17.9% [6]. However, peripheral and central NP together as the two fundamental conditions in pain is associated with serious social, psychological, and economic consequences. Anxiety, sleep disorders, and depression are common and severe in patients with NP, and patients' work time and the quality of life are significantly affected by NP as compared to the other conditions [7–9]. It is estimated that in the United States, the annual economic cost of chronic pain is at least $560–635 billion, including the incremental cost of health care ($261–300 billion) and the cost of lost productivity ($297–336 billion) [10]. A cohort study implementing the US health insurance claims database indicated that patients with NP are associated with an approximately 3-fold increase in healthcare costs as compared to those without NP [11]. The total cost of NP per patient was around €9,305–14,446 in Europe [9].

Multiple factors can sensitize nociceptors, such that no single pharmaceutical treatment is universally effective for PNP [2]. However, the potential therapeutic role of acupuncture and associated techniques in peripheral neuropathic pain (PNP) have been widely assessed experimentally and clinically exhibiting promising results [12–14]. Acupuncture and associated techniques are the most popular types of complementary alternative medicine available in China and the Western healthcare system and are believed to modulate local inflammatory reactions and the status of the whole body. Those techniques have been increasingly used to treat chronic pain related to PNP [15]. Currently, evidence about direct comparisons of clinical efficacy of those techniques on PNP and their associated cost-effectiveness assessment is still lacking. In this study, we conducted a two-step analysis. First, we performed a frequentist-based network meta-analysis to estimate the therapeutic effects of different acupuncture and moxibustion techniques. Second, a cost-effectiveness analysis (CEA) was performed to assess the economic feasibility of different acupuncture and moxibustion techniques.

## 2. Material and Methods

### 2.1. Network Meta-Analysis Process

The network meta-analysis was conducted according to Preferred Reporting Items for Systematic Reviews and Meta-Analyses guidelines for network meta-analysis (PRISMA-NMA) and acupuncture (PRISMA-A), and a published research protocol on PROSPERO (CRD: 42020203315).

#### 2.1.1. Search Strategies

The literature search was performed on the following electronic databases from inception to September 2020: Medline, Embase, Cochrane Library, Web of Science, China National Knowledge Infrastructure (CNKI), Wanfang database, WeiPu (VIP) database, and China Biology Medicine (CBM) database. The search terms included “acupuncture,” “electro-acupuncture,” “warming needling,” “fire needling,” “bloodletting,” “auriculo-acupuncture,” “moxibustion,” “cupping,” “collateral pricking,” “needle knife,” “neuropathic pain,” and “randomized controlled trial.” Titles and abstracts were screened by two authors independently; also, the bibliographies of articles were scanned for additional relevant studies.

#### 2.1.2. Eligibility Criteria

(1) Randomized controlled trials or cohort studies that fulfilled the diagnostic criteria for PNP were included [3]; (2) studies conducted on humans; (3) assessment of acupuncture/moxibustion techniques; (4) each arm in a study should include only one intervention for PNP; (5) the data provided in the articles should be sufficient to estimate the risk differences (RDs) and the corresponding 95% CIs in both groups. Moreover, studies on herbal drugs or their associated products should be excluded. Only the most recent publication was preserved if the previous studies were conducted on the same population. Duplicated studies of previously published data were excluded. Case-control studies, case reports/series, letters, reviews, editorials, and article comments were not suitable in this analysis and should be excluded.

#### 2.1.3. Outcome Measurements

The primary outcome was at least 20% relief of pain intensity. This could be assessed using pain evaluation scales (e.g., visual analogue scale, the McGill pain questionnaire, and symptoms scale) or pain threshold detectors.

#### 2.1.4. Data Collection

Two authors (Liu and Li) extracted data from original studies independently using a predesigned data extraction form. The following information was collected: first author's names, publication years, regions, ethnicities, sex, sample sizes, diagnoses, time of onset, interventions, treatment courses, and the number of drops. The discrepancies were resolved by referring to the original articles or consulting a superior author (Lin or Zheng).

#### 2.1.5. Assessment of Bias and Evidence Quality

The ROB 2.0 assessment tool was used to evaluate the risk of bias. The assessment of each study was carried out independently by two researchers (Liu and Li) in the process of data extraction. The differences in the data from the two researchers were solved through discussion and negotiation; if the contradiction could not be solved after negotiation, then a superior researcher (Zheng) was consulted.

#### 2.1.6. Network Meta-Analysis

Network meta-analysis was based on the frequentist method and calculated using the “netmeta” package (version 1.2-1) in R (version 3.6.3). The package constructed network models of direct or indirect comparisons of individual interventions. The heterogeneity of the treatment effects between studies was evaluated by the chi-square test of *Q*, *τ*^2^, and *I*^2^ metrics. A fixed-effects model for data synthesis was preferable if no heterogeneity was identified in the treatment effects between studies; otherwise, the random-effects model would be adopted. Data synthesis was carried out, and the treatment effects of interventions were examined (RDs and 95% CI) by comparing them to the reference treatment. For pairwise comparisons of the two treatments, a league plot of relative effect was presented. Sensitivity analysis was performed using a “split-node” method introduced by Dias et al. [16] to access the consistency between direct and indirect evidence. Publication bias was assessed through graphical inspection of the asymmetry of the funnel plot and evaluated by Egger, Begg–Mazumdar, and Thompson–Sharp methods.

### 2.2. Cost-Effectiveness Analysis

#### 2.2.1. Referencing Resources

The cost of acupuncture and moxibustion treatments was extracted from a government document (Table 1. The reference prices of pharmaceutical medications were evaluated based on the information on the drugs retrieved from the local healthcare service pricing system (Table 2).

#### 2.2.2. Cost Estimation

Net outpatient treatment costs, including direct and indirect, were estimated in a 1-week treatment period. The indirect costs include lost working income and the corresponding transportation expenses. The average income of citizens in Guangzhou city is 61,241 RMB (Chinese Yuan)/year in 2018 [17] (about 22.85 RMB/hour in an 8 h working period of a day). The transportation expense was estimated by assuming the use of public transport by the patients as 8 RMB each time they came to the clinic. We also assumed the patient spent 2 h on the road and another hour for the interview and treatment. The net indirect cost was 76.55 RMB every time a patient visited. For pharmaceutical treatments, the indirect cost was considered once a week for one-week replenishment in ordinary clinical settings. For nonpharmaceutical treatments, indirect costs should be incorporated into the costs per visit.

#### 2.2.3. Cost-Effectiveness Assessment

The number needed to treat (NNT) values of each intervention were calculated by obtaining the reciprocal of the RDs synthesized in the network meta-analysis. Successively, the cost-effectiveness of the intervention was evaluated as incremental cost per additional responder (ICPR) as compared to a reference treatment that could be acquired by multiplying the NNTs with direct or total costs of the treatments. A smaller ICPR of the treatment indicated that it costs less to one extra responder, and thus, the treatment would be cost-effective.

## 3. Results

### 3.1. Characteristics of Studies

**Figure 1:**
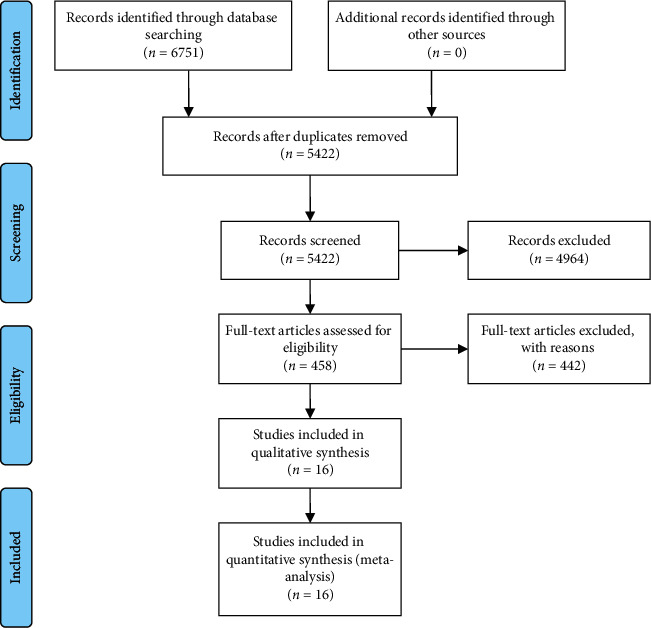
Flowchart of the selection process.

A total of 6751 studies were retrieved in the preliminary literature search. Of these, 16 studies [18–33] that satisfied the eligibility criteria were identified, and 1308 participants were included in the current study. The selection process of the studies was illustrated in Figure 1. All the included studies were conducted in China. The etiology that causes the PNP in the eligible studies includes sciatica, cervical spondylotic radiculopathy, shingles, postherpetic neuralgia, occipital neuralgia, cervical entrapment syndrome, and trigeminal neuralgia. The pain intensity was evaluated using scales or pain threshold detection. The characteristics of the included studies are summarized in Table 3.

### 3.2. Risk of Bias

**Figure 2:**
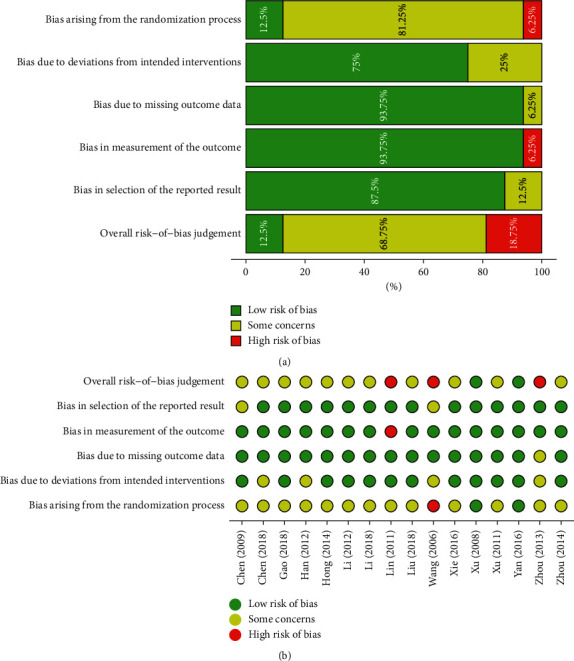
Risk of bias judgment in each domain (a) and risk of bias rating of individual study (b).

The potential risks of bias were examined. The risk of bias in each domain and the bias rating of the individual study are depicted in Figure 2. The results indicated that randomization processes were the major sources of bias. The overall risk-of-bias judgment was as follows: 12.5% of the studies were at a low risk of bias, 68.75% of the studies had some concerns about the risk of bias, and the remaining 18.75% were at a high risk of bias.

### 3.3. Network Construction

**Figure 3:**
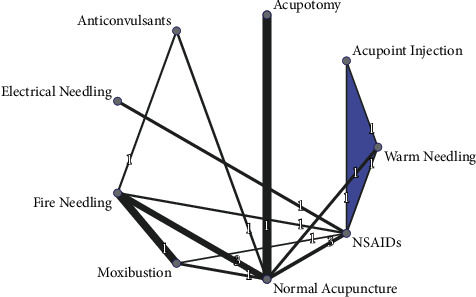
The network of direct comparisons of the treatments. Lines connecting two treatments indicate direct comparisons for those two arms of treatments. The width of the lines represents the inversion effect estimates. The blue shading area infers multiple arms of direct comparisons in a single study. NSAIDs, nonsteroidal anti-inflammatory drugs.

The network of comparisons included 1 three-arm trial and 15 two-arm trials. Acupuncture and moxibustion techniques, such as normal acupuncture, acupoint injection, acupotomy (round-sharp needling), electrical needling, fire needling, moxibustion, and warm needling, were included in the current study. We also classified the pharmaceutical treatments into two categories, namely, anticonvulsants and nonsteroidal anti-inflammatory drugs (NSAIDs). The treatment effects are synthesized based on direct and indirect pairwise comparisons. The effects of NSAIDs were treated as a reference. The network correlation of a graphical indicator is shown in Figure 3.

### 3.4. Data Synthesis

**Figure 4:**
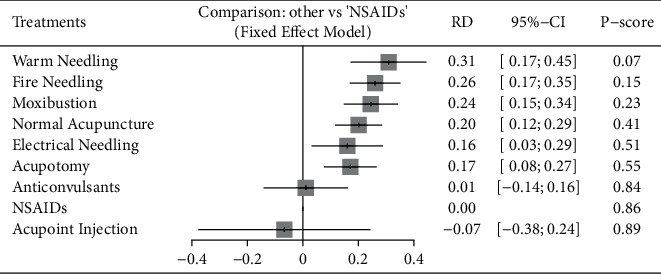
Forest plot of treatment effects vs. NSAIDs (risk difference). The smaller *P*-score value indicates a better effect ranking. NSAIDs, nonsteroidal anti-inflammatory drugs; RD, risk difference; 95% CI, 95% confidence interval.

**Figure 5:**
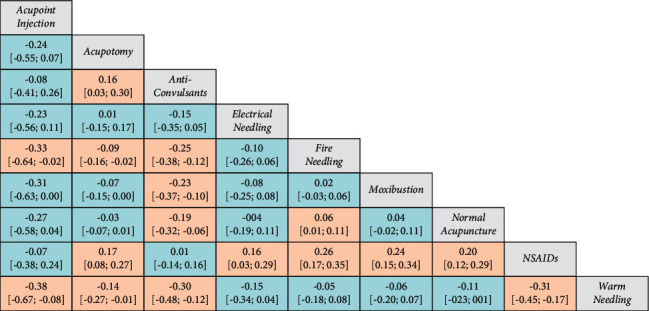
League plot of relative effects (RDs) among nine interventions. The treatment in the corresponding column is compared to the treatment in the corresponding row. Values are the RDs and related 95% CI. Bold fonts and orange-shaded cells represent significant relative effects (*P* < 0.05).

The overall heterogeneity *Q* metric was 12.49 (*P* = 0.1873). Besides, the *τ*^2^ metric was 0.0024 and the *I*^2^ metric of the heterogeneity was 27.9% (95% CI: 0.0–65.4%). No heterogeneity was identified; thus, a fixed-effects model was used to estimate the pooled effect size for the RD of each treatment against the effect of NSAIDs as the reference (Figure 4). The effects of the treatment were scored as *P* values. In the current case, a small *P*-score indicates an improved treatment effect. Pairwise comparisons of the relative treatment effects were presented as a league plot (Figure 5). Typically, all the included acupuncture and moxibustion techniques showed superior improvements in PNP relief as compared to NSAIDs (*P* < 0.05), except acupoint injection. Anticonvulsants showed stronger effects (RD: 0.01 [−0.14; 0.16]) than NSAIDs; nevertheless, the effects were not significant (*P* > 0.05). Among the acupuncture and moxibustion techniques, warm needling (RD: 0.31 [0.17; 0.45]),6 fire needling (RD: 0.26 [0.17; 0.35]), and moxibustion (RD: 0.24 [0.15; 0.34]) were the most effective, but no significant differences were detected in the effects between these three interventions (*P* > 0.05).

### 3.5. Sensitivity Analysis

**Figure 6:**
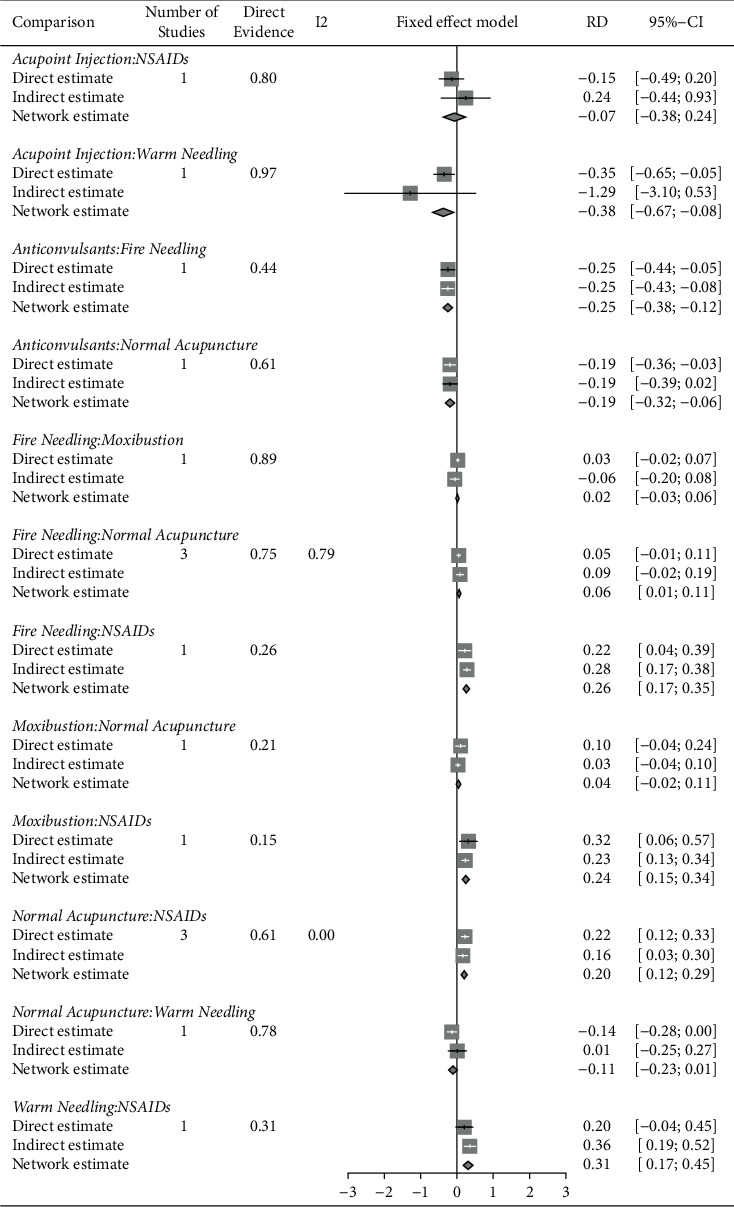
Split-node plot of treatment pairs.

The split-node plot in Figure 6 shows the consistency between direct and indirect evidence, which indicated the robustness of our network results. However, there was only one direct comparison for each electrical needling (vs. NSAIDs) and acupotomy (vs. normal acupuncture), and estimated effects of these isolated nodes depend merely on the direct evidence, which the “split-node” method is incapable to assess.

### 3.6. Publication Bias

**Figure 7:**
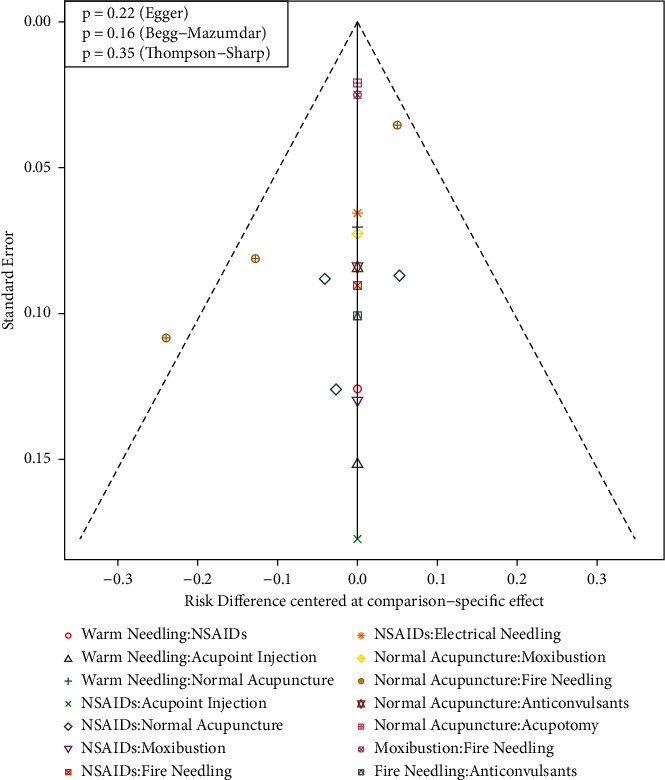
Funnel plot of comparison-specific effects of treatment pairs. Symbols indicate comparisons of different treatment pairs with an indicator on the upper-right corner; *P* values of statistical tests for publication bias are shown on the upper-left corner. NSAIDs, nonsteroidal anti-inflammatory drugs.

The funnel plot was used to assess the publication bias (Figure 7), which showed the symmetry distribution of the comparison-specific effects of treatment pairs. Further tests of Begg, Egger–Mazumdar, and Thompson–Sharp tests showed no potential publication bias was identified (*P* > 0.05).

### 3.7. Cost-Effectiveness Analysis

The NNT exhibits one additional pain relief responder relative to NSAIDs, which can be calculated from the inversion of RDs (Table 4). In the situation when the treatment effect does not differ significantly from the reference, the zero is inevitably included in the 95% CI. Consequently, the NNT becomes infinite, containing two disjoint regions. To emphasize the continuity, we described the 95% CI of such a treatment effect in the format of “number needed to harm” to ∞ to “NNT” as recommended in a previous study [34]. The costs of treatments in 1 week are estimated in Table 5.

**Figure 8:**
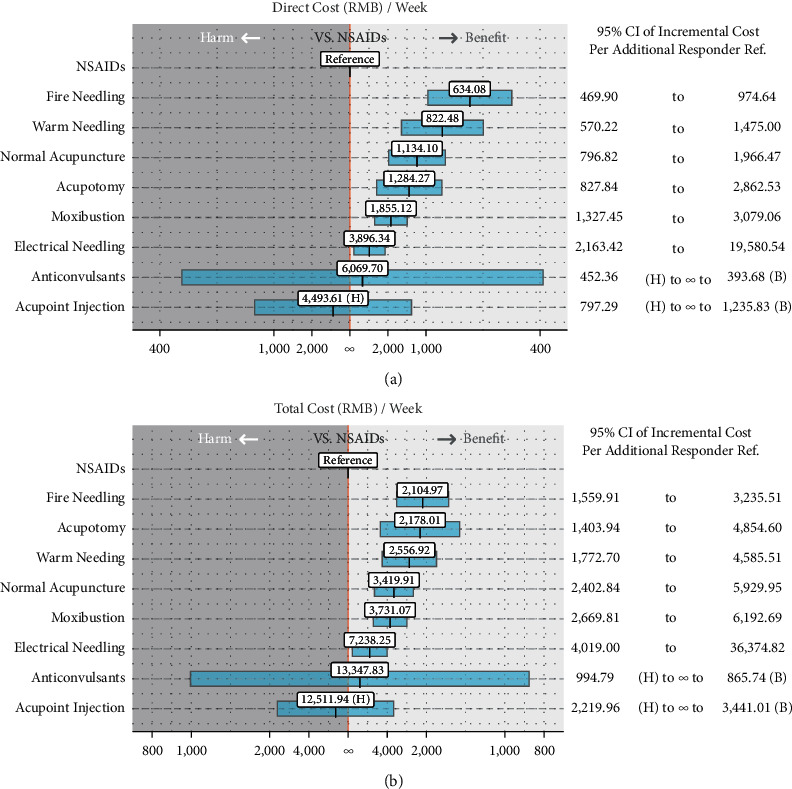
Incremental direct (a) and total (b) costs per additional responder related to NSAIDs in the 1-week treatment period. The treatment in the corresponding column is compared to the treatment in the corresponding row. Values are the RDs and related 95% CI. Bold fonts and orange-shaded cells represent significant relative effects (*P* < 0.05). 95% CI, 95% confidence interval; Ref., reference cost (NSAIDs); (H), cost to be harmful; (B), cost to be beneficial.

In the CEA of direct costs (Figure 8(a)), fire needling had the lowest ICPR (ICPR: 634.08 [469.90; 974.64] RMB/week) relative to NSAIDs, while that for warm needling was 822.48 (570.22; 1,475.00) RMB/week, normal acupuncture was 1,134.10 (796.82; 1,966.47) RMB/week, acupotomy was 1,284.27 (827.84; 2,862.53) RMB/week, moxibustion was 1,855.12 (1,327.45; 3,079.06) RMB/week), and electrical needling was 3,896.34 (2,163.42; 19,580.54) RMB/week).

Similar results are shown in the CEA of total costs (Figure 8(b)). Fire needling still had the lowest ICPR (2,104.97 [1,559.91; 3,235.51] RMB/week) relative to NSAIDs, followed by acupotomy (ICPR: 2,178.01 [1.403.94; 4,894.60] RMB/week), warm needling (ICPR: 2,556.92 [1,772.70; 4,585.51] RMB/week), normal acupuncture (ICPR: 3,419.91 [2,402.84; 5,929.95] RMB/week), moxibustion (ICPR: 3,731.07 [2,669.81; 6,192.69] RMB/week), and electrical needling (ICPR: 7,238.25 [4,019.00; 3,6374.82] RMB/week).

Conversely, there is indiscriminate harm or benefit of (in)direct ICPR in anticonvulsants and acupoint injection relative to NSAIDs. Patients might not benefit significantly from anticonvulsants and acupoint injection as compared to NSAIDs.

## 4. Discussion

We presented a comprehensive systematic review and network meta-analysis that focuses on acupuncture and moxibustion treatments for PNP. Herein, seven widely applicable acupuncture and moxibustion techniques and two categories of pharmaceutical therapies have been assessed. However, NSAIDs, one of the included categories of pharmaceutics in the present study, are not recommended in the guidelines, but are still prescribed in Chinese clinics. Nonetheless, 6 of the included studies involved NSAID treatments. Since analgesics, such as NSAIDs have only ceiling effect against the pain of neuropathic origin, they constitute the reference group in our analyses. Furthermore, techniques applying heat, such as warm needling, fire needling, and moxibustion, showed the highest therapeutic rankings in our network meta-analysis. Except for acupoint injection, we observed that the acupuncture and moxibustion techniques exhibited higher efficacy as compared to pharmaceutics. Previous meta-analyses have shown controversial results. Dimitrova et al. [37] concluded that acupuncture is beneficial in some peripheral neuropathies, while Ju et al. [38] speculated that the data are insufficient to conclude the effects of acupuncture for PNP. Despite different primary outcomes and diverse conclusions, both studies emphasized the demand for additional rigorously designed trials to clarify the actual therapeutic effects of acupuncture in PNP treatment. Similarly, the included trials, with diverse qualities, in the current study were exclusively conducted in China. The risk-of-bias assessment addressed some of the great concerns of the risk of bias, among which randomized processes were the major sources of bias.

A secondary analysis of the assessment of the cost-effectiveness correlation was performed among the included treatments. Based on the perspective of healthcare providers, at least one visit per week is necessary for acupuncture and moxibustion treatment, while one visit a week for the replenishment of pharmaceutical drugs is feasible in most clinical settings. Hence, the cost-effectiveness of different treatments was assessed in a 1-week treatment period. We found that fire needling is the most cost-effective treatment for PNP, especially for direct costs. Accounting for the total cost of each treatment from the perspective of patients' treatment burdens, fire needling still exhibited maximal cost-efficiency. Notably, acupotomy became the second-ranked cost-efficient therapy in our CEA of total costs that required only a few visits per week. PNP is a chronic condition that needs prolonged treatment. Potential risks of adverse effects and abuse concerns should be tagged, especially in the second and third-line therapies for PNP containing opiates or neurotoxin [39]. Acupuncture and moxibustion techniques are widely used in pain management, which cause fewer side effects than other pharmaceutical alternatives. Our results suggested that patients would have better responses to acupuncture and moxibustion treatments in lowering pain intensity. The clinical and economic feasibility of therapy is a critical factor for healthcare providers and patients. We also found that fire needling was the most cost-effective among the included therapies.

We have assessed a total of 7 widely applicable acupuncture and moxibustion techniques that elaborate specific manipulations to achieve their clinical effects. Normal acupuncture is the most fundamental acupuncture technique that simply inserts fine needles (acupuncture needles) into the skin or acupoints. Sensations of soreness, heaviness, numbness, or distension around the insertion points through the twisting of the body of the needles, a process called “deqi,” are believed to increase the therapeutic effects [30]. Greater effects are often achieved by applying additional sources of stimulations on the needles, such as electrical currents or heats, namely, electrical needling and warm needling. In electrical needling, micro-electric currents are applied on the needle resulting in circuit loops formation and subsequent stimulations on the needling sites [31]. Warm needling is a combination of acupuncture and moxibustion techniques. A burning small moxa tower affixed on the handle of the acupuncture needle, and heat generated from the burning moxa would radiate superficially or be conducted through the needle body to between tissues underneath the skin [32]. Fire needling involves a slightly different technique compared to normal acupuncture, that is, rapid insertion of red-hot specialized needles that are made of tungsten-manganese alloy into acupoints and lift without leaving the needles on the insertion points [33]. Acupoint injection is a kind of therapeutic method that injects medicine or nutrient supplements into acupoints subcutaneously or intramuscularly [34]. The moxibustion technique involves stimulating acupoints or areas of the body by radiating heat and volatile chemicals released from a burning moxa stick [35]. Acupotomy (round-sharp needling) is developed from an ancient acupuncture technique that integrates modern anatomy and sports medicine theories. A needle with a sharp round tip was inserted deeply into the skin, and by manipulating the sharp edge at the end of the needle tip, compressions or adhesions can be resolved [36].

Diagnosis and assessment of NP are challenging due to the absence of pain biomarkers. The diagnosis of NP rarely relies on other diagnosis techniques except for clinical criteria, and the crucial point is to differentiate pain caused by nerves lesion from other types of pain. In our current study, the assessment of pain is based on tools for common situations such as visual analogue scale, short-form McGill pain questionnaire, pain threshold, and symptom scales. However, as stated before, apart from pain, NP or PNP patients are often accompanied by psychological conditions and lower quality of life. Future research should emphasize whether acupuncture and the associated techniques can improve the psychological status and the quality of life among NP patients. Screening tools that are specialized for assessing NP and the associated psychometric properties. Many of them are available, like the Leeds assessment of neuropathic symptoms and signs, neuropathic pain questionnaire, PainDetect, ID-Pain, and DN4, which were developed and validated [4].

The primary limitation of the present study is the small number of studies that fulfill the selection criteria and small sample sizes of the included studies, leading to insufficient direct comparisons between treatment pairs in the network construction. Agents, such as gabapentinoid, tricyclic antidepressants, and norepinephrine reuptake inhibitors, are currently recommended as first-line analgesics for NP management [40]. The efficacy of anticonvulsants was indifferent to the efficacy of NSAIDs on PNP and might be underestimated in the present analysis, and more upcoming researches are needed to be included to produce more precise inferences. There was only one direct comparison for each electrical needling and acupotomy therapy and the robustness of their network effects should be interpreted with cautions; further studies on different comparisons about these two therapies are warranted. Our network meta-analysis involved only mono-therapy interventions. Herein, we did not assess the treatments and additive effects of combination therapies of acupuncture and moxibustion with other therapies, which might be a common clinical application. Moreover, studies on PNP due to various causes and different disease duration and treatment periods were also included in this meta-analysis. These might introduce sources of confounding to our results.

## 5. Conclusion

In summary, our findings revealed that approaches for easing PNP including acupuncture and moxibustion techniques are cost-effective as compared to pharmaceutics except for acupoint injection. The current results also suggested that acupuncture and moxibustion techniques involving heat modalities provide preferable treatment responses in PNP relief.

## Figures and Tables

**Table 1:** Price and dosage of acupuncture and moxibustion treatments.

Treatment	Price unit	Price (RMB)	Dosage	Treatment frequency (times a week)	Notes
Normal acupuncture	5 acupoints	18.98	10 acupoints	6	Charge 3.8 RMB for each extra acupoint
Acupoint injection	2 acupoints	16.50	5 acupoints	7	Charge 8.8 RMB for each extra acupoint
Acupotomy	1 site	55.00	2 sites	2	—
Electrical needling	2 acupoints	15.75	16 acupoints	7	Charge 10.5 RMB for every 2 extra acupoints
Fire needling	3 acupoints	16.50	6 acupoints	5	Charge 5.5 RMB for each extra acupoint
Moxibustion	2 acupoints	25.30	10 acupoints	6	Charge 6.3 RMB for each extra acupoint
Warm needling	5 acupoints	33.00	10 acupoints	7	Charge 3.3 RMB for every 5 extra acupoints

**Table 2:** Price and dosage of pharmaceutical medications.

Medication	Category	Specification	Dosage	Price (RMB)^*∗*^
Diclofenac diethylamine emulgel	NSAIDs	20 g	2 g qid	17.18
Ibuprofen sustained release capsules	NSAIDs	0.3 g	0.3 g bid	0.65
Indometacin enteric-coated tablets	NSAIDs	25 mg	25 mg tid	0.15
Nabumetone capsules	NSAIDs	0.25 g	1 g qd	1.28
Nimesulide dispersible tablets	NSAIDs	0.1 g	0.1 g bid	1.73
Carbamazepine tablets	Anticonvulsants	200 mg	200 mg tid	0.84
Gabapentin capsules	Anticonvulsants	100 mg	1200 mg tid	0.46

**Table 3:** Characteristics summary of included studies.

Author	Region	Diagnosis	Assessment tool	Arms	Intervention	Pain duration	Participants	Drop	Female/male	Responder/total	Treatment duration
Chen et al. [18]	China	Sciatica	Pain threshold	3	G1: warm needling	G1: 5.25 ± 3.59 y	G1: 30	G1: 0	G1: 8/22	G1: 27/30	10 d
					G2: nimesulide dispersible tablets	G2: 5.78 ± 4.87 y	G2: 30	G2: 0	G2: 9/21	G2: 22/30	
					G3: acupoint injection	G3: 4.71 ± 3.96 y	G3: 30	G3: 0	G3: 10/20	G3: 19/30	

Chen et al. [19]	China	PHN	VAS	2	G1: normal acupuncture	G1: 1.35 ± 0.37 y	G1: 45	G1: 0	G1: 16/29	G1: 36/45	20 d
					G2: fire needling	G2: 1.42 ± 0.39 y	G2: 45	G2: 0	G2: 14/31	G2: 43/45	

Gao [20]	China	Shingles	VAS	2	G1: electrical needling	G1: 3.32 ± 1.21 y	G1: 40	G1: 0	G1: 16/24	G1: 40/40	10 d
					G2: indometacin enteric-coated tablets	G2: 3.12 ± 1.43 y	G2: 40	G2: 0	G2: 15/25	G2: 34/40	

Han [21]	China	Cervical entrapment syndrome	VAS	2	G1: acupotomy (round-sharp needling)	G1: 3 m–11 y	G1: 68	G1: 0	G1: 27/41	G1: 66/68	14 d
					G2: normal acupuncture	G2: 5 m–12 y	G2: 67	G2: 0	G2: 28/39	G2: 67/67	

Hong [22]	China	Occipital neuralgia	VAS	2	G1: fire needling	G1: 3.8 ± 1.6 d	G1: 30	G1: 0	G1: 16/14	G1: 30/30	5 d
					G2: carbamazepine tablets	G2: 3.7 ± 1.8 d	G2: 27	G2: 0	G2: 15/12	G2: 21/27	

Li et al. [23]	China	PHN	VAS	2	G1: moxibustion	G1: 5.90 ± 1.44 m	G1: 40	G1: 0	G1: 17/23	G1: 39/40	30 d
					G2: fire needling	G2: 6.07 ± 1.27 m	G2: 40	G2: 1	G2: 18/22	G2: 39/39	

Li [24]	China	PHN	VAS	2	G1: indometacin enteric-coated tablets	G1: 4.05 ± 2.13 m	G1: 42	G1: 0	G1: 15/27	G1: 28/42	15 d
					G2: normal acupuncture	G2: 4.13 ± 2.22 m	G2: 42	G2: 0	G2: 16/26	G2: 36/42	

Lin et al. [25]	China	PHN	VAS	2	G1: moxibustion	G1: 5.90 ± 1.44 m	G1: 30	G1: 1	G1: 13/17	G1: 28/29	28 d
					G2: ibuprofen sustained release capsules	G2: 6.07 ± 1.27 m	G2: 30	G2: 3	G2: 16/14	G2: 19/27	

Liu [26]	China	PHN	VAS	2	G1: indometacin enteric-coated tablets	G1: 4.11 ± 1.69 m	G1: 40	G1: 0	G1: 18/22	G1: 32/40	15 d
					G2: normal acupuncture	G2: 4.12 ± 1.61 m	G2: 40	G2: 0	G2: 17/23	G2: 38/40	

Wang et al. [27]	China	PHN	VAS	2	G1: fire needling	G1: 1–10 m	G1: 28	G1: 0	G1: 12/16	G1: 28/28	14 d
					G2: normal acupuncture	G2: 1–11 m	G2: 28	G2: 0	G2: 13/15	G2: 28/28	

Xie and Chen [28]	China	Trigeminal neuralgia	VAS	2	G1: carbamazepine tablets	G1: 9.8 ± 2.6Y	G1: 63	G1: 0	G1: 28/35	G1: 47/63	1 m
					G2: normal acupuncture	G2: 9.4 ± 2.7 y	G2: 63	G2: 0	G2: 27/36	G2: 57/63	

Xu and Kang [29]	China	Occipital neuralgia	SF-MPQ	2	G1: moxibustion	NA	G1: 20	G1: 0	NA	G1: 20/20	10 d
					G2: normal acupuncture		G2: 20	G2: 0		G2: 18/20	

Xu [30]	China	PHN	VAS	2	G1: fire needling	26 d–3 y	G1: 44	G1: 0	Total: 35/52	G1: 41/44	10 d
					G2: normal acupuncture		G2: 43	G2: 0		G2: 30/43	

Yan et al. [31]	China	PHN	VAS	2	G1: fire needling	G1: 180.41 ± 25.71 d	G1: 38	G1: 0	G1: 17/21	G1: 37/38	28 d
					G2: diclofenac diethylamine emulgel	G2: 175.50 ± 26.05 d	G2: 37	G2: 0	G2: 17/20	G2: 29/37	

Zhou [32]	China	Occipital neuralgia	VAS	2	G1: normal acupuncture	G1: 4.13 ± 1.22 d	G1: 60	G1: 2	G1: 33/27	G1: 55/58	5 d
					G2: nabumetone capsules	G2: 3.87 ± 1.39 d	G2: 60	G2: 5	G2: 29/31	G2: 40/55	

Zhou et al. [33]	China	Cerical spondyliotic radicuolathy	Symptom scales	2	G1: warm needling	NA	G1: 30	G1: 0	G1: 8/22	G1: 30/30	10 d
					G2: normal acupuncture		G2: 30	G2: 0	G2: 12/18	G2: 26/30	

PHN, postherpetic neuralgia; VAS, visual analogue scale; SF-MPQ, short-form McGill pain questionnaire; NSAIDs, nonsteroidal anti-inflammatory drugs; G1 or (2, 3), group 1 or (group 2, group 3); NA, not available; d, day; m, month; y, year.

**Table 4:** Risk difference and number needed to treat for each intervention.

Treatments	RD [95% CI]		NNT [95% CI]	
NSAIDs	Reference		Reference	
Normal acupuncture	0.20	[0.12; 0.29]	4.98	[8.63; 3.50]
Anticonvulsants	0.01	[−0.14; 0.16]	95.08	[7.09 (H) to ∞ to 6.17(B)]
Acupoint injection	−0.07	[−0.38; 0.24]	−14.96	[2.65 (H) to ∞ to 4.12 (B)]
Acupotomy	0.17	[0.08; 0.27]	5.84	[13.01; 3.76]
Electrical needling	0.16	[0.03; 0.29]	6.24	[31.34; 3.46]
Fire needling	0.26	[0.17; 0.35]	3.84	[5.91; 2.85]
Moxibustion	0.24	[0.15; 0.34]	4.08	[6.78; 2.92]
Warm needling	0.31	[0.17; 0.45]	3.24	[5.80; 2.24]

NSAIDS, nonsteroidal anti-inflammatory drugs; RD, risk difference; NNT, number needed to treat; 95% CI, 95% confidence interval; (H), number needed to treat to be harmful; (B), number needed to treat to be beneficial.

**Table 5:** Treatment costs in a one-week period.

Treatments	Direct cost/week (RMB)	Total cost/week (RMB)	Average cost/week (RMB)
*Nonpharmaceutical treatments*			
Normal acupuncture	227.88	687.18	
Acupoint injection	300.30	836.15	
Acupotomy	220.00	373.10	
Electrical needling	624.75	1160.60	
Fire needling	165.00	547.75	
Moxibustion	454.20	913.50	
Warm needling	254.10	789.95	

*Pharmaceutical treatments*			
NSAIDs			Direct: 24.08
			Total: 100.63
Diclofenac diethylamine emulgel	48.10	124.654	
Ibuprofen sustained release capsules	9.10	85.65	
Indometacin enteric-coated tablets	3.15	79.70	
Nabumetone capsules	35.84	112.39	
Nimesulide dispersible tablets	24.22	100.77	
Anticonvulsants			Direct: 63.84
			Total: 140.39
Carbamazepine tablets	11.76	88.31	
Gabapentin capsules	115.92	192.47	

NSAIDs, nonsteroidal anti-inflammatory drugs.

## Data Availability

All data supporting our findings are available within the manuscript.

## Abbreviations

NP: Neuropathic pain
PNP: Peripheral neuropathic pain
CEA: Cost-effectiveness analysis
RD: Risk difference
CI: Confidence interval
NNT: Number needed to treat
ICPR: Incremental cost per additional responder
NSAIDs: Nonsteroidal anti-inflammation drugs.
